# International health library associations urge the ICMJE to seek information specialists as peer reviewers for knowledge synthesis publications

**DOI:** 10.5195/jmla.2021.1301

**Published:** 2021-07-01

**Authors:** Sandy Iverson, Maurella Della Seta, Carol Lefebvre, Ann Ritchie, Lisa Traditi, Kevin Baliozian

**Affiliations:** 1 President, The Canadian Health Libraries Association (CHLA/ABSC); 2 President, The European Association for Health Information and Libraries (EAHIL); 3 MLA Representative to EAHIL; 4 National Manager, The Australian Library and Information Association/Health Libraries Australia (ALIA-HLA); 5 President, US Medical Library Association (MLA); 6 Executive Director, MLA

**To the International Committee of Medical Journal Editors (ICMJE)**

Dear Colleagues,

We are writing to you to encourage journal editors to actively seek information specialists as peer reviewers for knowledge synthesis publications and to advocate for the recognition of their methodological expertise.

Evidence indicates that few systematic review and other knowledge synthesis publications reflect the participation of information specialists [1–4] despite the recommendations of international knowledge synthesis organizations such as the Campbell Collaboration, Cochrane, and the Joanna Briggs Institute [5–7]. There is also a growing body of research suggesting that there is a crisis in the reproducibility of methods reported in these types of publications [2, 8–10]. This is the case despite reporting guidelines like PRISMA having been widely known for a decade [11] and the benefits of information specialists' involvement in the conduct of systematic and scoping reviews having been well documented [1, 3, 12].

Based on our extensive collective international experience and the published evidence, it is our view that journal editors should more actively recruit information specialists as peer reviewers for knowledge synthesis publications. Information specialists bring to the table a unique set of skills, including considerable methodological expertise that can help address issues of rigor and research waste [13]. In the same way that inappropriate data collection methods for primary research undermine the integrity of research results and conclusions, the quality of the search—the data collection method for reviews—can undermine the integrity of a systematic review. Without robust and thoroughly critiqued methods for identifying studies for inclusion, knowledge syntheses are subject to potential error and systematic bias. To this end, information specialists are encouraged to ensure that the search strategies for systematic reviews and other knowledge synthesis publications are reviewed by a second expert searcher prior to finalizing the study identification process [14]. This is supplemental to the other aspects of the peer reviewing process that occur immediately prior to publication.

The membership of the associations contributing to this letter represent the most skilled, qualified, and experienced expert searchers in the fields of medicine and health care in the world. They are deeply invested in improving the quality of knowledge synthesis publications. These health library associations encourage their members to register as potential peer reviewers for journals in their specialty areas. A recent survey of librarians and information specialists, however, suggests that these professionals are rarely approached to participate in the peer review of systematic reviews or their search strategies at the publication stage [15]. We note that the selection of peer reviewers prior to publication is the responsibility of journal editors, as described in the ICMJE recommendations section II.C.2.c(16), and peer review plays a crucial role in maintaining the quality and trustworthiness of research publications. To this end, journal editors can solicit information specialists to peer review knowledge synthesis search strategies by contacting association leadership for recommendations, by reaching out through professional networks, and via social media.

We ask, therefore, that the ICMJE should recommend to their journal editors that information specialists be approached for methodological peer review. To assist with this, you may find the Librarian Peer Reviewer Database (https://sites.google.com/view/mlprdatabase/home) of assistance. This database was created by a group of professional librarians to connect experts in systematic searching with journal editors seeking their input in the peer review process.
